# Effectiveness and Safety of Contrast Cryolipolysis for Subcutaneous-Fat Reduction

**DOI:** 10.1155/2018/5276528

**Published:** 2018-11-29

**Authors:** Marília Bueno Savacini, Débora Tazinaffo Bueno, Ana Carolina Souza Molina, Ana Caroline Almeida Lopes, Caroline Nogueira Silva, Renata Gomes Moreira, Stephani Almeida, Renata Michelini Guidi, Estela Sant'Ana, Richard Eloin Liebano

**Affiliations:** ^1^Ibramed Center for Education and Advanced Training (CEFAI), Amparo, Brazil; ^2^Clinical Laboratory of the Ibramed Center for Education and Advanced Training (CEFAI), Amparo, Brazil; ^3^Research, Development & Innovation Department IBRAMED, Ibramed Research Group (IRG), Amparo, Brazil; ^4^Human Development and Technologies. Universidade Estadual Paulista (UNESP), Brazil; ^5^Electrical Engineering Department, Faculty of Medical Sciences, University of Campinas (Unicamp), Brazil; ^6^Department of Physiotherapy, Federal University of São Carlos, São Carlos, Brazil

## Abstract

Cryolipolysis is the noninvasive treatment of localized fat through cold-induced panniculitis. The purpose of the present study was to evaluate the safety and efficacy of contrast cryolipolysis for subcutaneous-fat reduction. Contrast cryolipolysis mixes the principles of conventional cryolipolysis and periods of heating in accordance with the contrast lipocryolysis process. Twenty-one subjects aged 34 ± 9 years were treated with contrast cryolipolysis in the regions of abdomen and flanks through the Polarys® device. Anthropometry, standardized photographs, measurements with a skinfold caliper, and diagnostic ultrasounds were performed at the baseline and during follow-ups at 30, 60, and 90 days after the treatment. The safety assessments included laboratory testing and monitoring of the adverse events. The level of significance for all tests was set at* P *< 0.05. No significant differences in weight and body mass index were found. The waist measurements at the baseline and 30-day follow-up had significant differences, as did the measurements at the 30-day and 60-day follow-ups. The skinfold and ultrasound measurements were significantly reduced in the treated areas in all the time points compared to the baseline. The laboratory results showed no significant changes from baseline. Temporary adverse effects were resolved spontaneously. This study confirmed that contrast cryolipolysis is safe and effective in reducing the fat layer and improving body contouring.

## 1. Introduction

Localized adiposity is an abnormal accumulation of fat in usual anatomical locations, and it is an important unaesthetic condition [1]. Liposuction has always been considered the standard treatment for body contouring; however, because of the potential complications associated with this procedure, new treatments were developed [2, 3]. Several treatments—including ultrasound, radiofrequency, and mesotherapy—have been developed to achieve adipocyte destruction [4–9]. Each technology employs a different mechanism to cause the apoptosis or necrosis of the targeted adipocytes.

**Figure 1:**
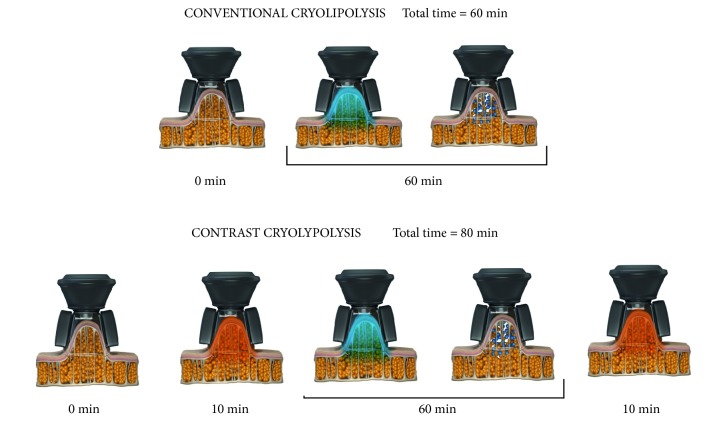
A comparison between conventional cryolipolysis and contrast cryolipolysis.

In recent years, a new technology for the noninvasive treatment of localized fat through cold-induced panniculitis—called cryolipolysis—appeared. This method is based on the concept that lipid-rich tissues are more susceptible to injury through cold than the surrounding water-rich tissue is [10]. On the other hand, studies [11–13] have shown that, when alternating low temperatures and cycles of heating, the lipids in the adipocytes crystallize more easily, which is similar to what happens in the tempering process of the food industry, and this process may improve the clinical outcome of the treatment. This method is known as contrast lipocryolysis. Based on both models (cryolipolysis and contrast lipocryolysis), a novel technology was conceived: contrast cryolipolysis. This technology differs from conventional cryolipolysis because it uses heating and cooling periods, and it differs from contrast lipocryolysis because it uses lower temperatures during cooling (Figure 1).

Although a large number of published studies on conventional cryolipolysis exist [14–24], studies assessing the effects of contrast cryolipolysis are still scarce. Therefore, the purpose of the present study was to evaluate the safe and efficacy of this method.

## 2. Materials and Methods

### 2.1. Subjects

This study included 21 healthy subjects aged between 18 and 50 years. The subject inclusion criteria were the presence of localized subcutaneous fat in the abdominal and flank regions and a body mass index (BMI) < 30. The subjects were excluded if they were in aesthetic treatment, had received some kind of treatment in the abdominal region in the 6 months before the start of this study, were pregnant or had experienced a recent pregnancy (within the past 6 months), or had a known history of cryoglobulinemia, cold urticaria, or paroxysmal cold hemoglobinuria.

**Figure 2:**
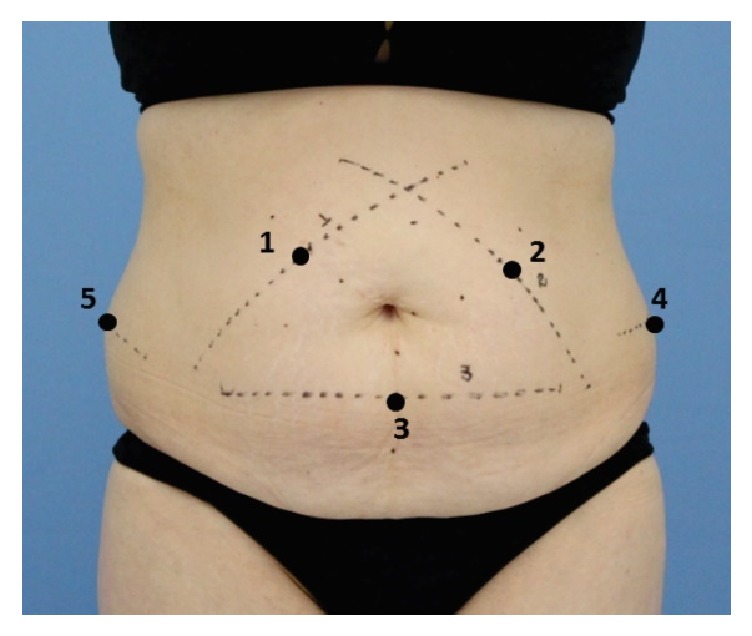
Points of evaluation and marked areas for the treatment. Each subject was treated at 1 or more areas for a total of up to 5 treatment areas; the areas were treated with either the medium or large applicator, based on the size of the localized fat area.

The decision of which region to treat (i.e., the abdomen or the flanks) was made according to individual needs (Figure 2).

Assessments of body composition were performed at the baseline and during follow-ups 30, 60, and 90 days after the treatment.

### 2.2. Ethical Aspects

The Research Ethics Committee: Institutions, Teaching, and Research approved this study: UNISEPE: CAAE: 61499416.5.0000.5490 (http://plataformabrasil.saude.gov.br). All subjects signed informed consent forms, and the treatment was performed by trained physiotherapist in the Clinical Laboratory of the Ibramed Center for Education and Advanced Training CEFAI (Amparo, São Paulo, Brazil).

### 2.3. Sample Size

The sample size was calculated in consideration of a difference of 2.0 mm in the adipose layer, as evaluated by ultrasound. An estimated standard deviation of 2.0 mm was also considered, based on data from a previous study [25] on the effects of cryolipolysis on fat in the abdominal region of women. For a level of significance of 0.05 and power of 80%, the Minitab software calculated that 17 participants would be required (Minitab, v.17, StateCollege, PA). Thus, in order to avoid possible sample losses that would interfere with data analysis, 21 patients were recruited.

### 2.4. Treatment Protocol

The subcutaneous-fat layer in the regions of the abdomen and flanks was treated with contrast cryolipolysis. It was heated to 40°C for 10 minutes, cooled for 60 minutes (−8°C), and heated again to 40°C for 10 minutes with the cryolipolysis device Polarys® (Ibramed, Indústria Brasileira de Equipamentos Médicos EIRELI).

The subjects were treated at 1 or more areas for a total of up to 5 treatment areas in 1 or 2 sessions. Areas were treated with either the medium or large applicator, based on the size of the localized fat area and the anatomical limitations of the applicator placement. The treatment sessions were performed with the subjects comfortably positioned in the dorsal decubitus position with a 45° stretcher inclination or in the lateral decubitus position. The curved vacuum applicator was positioned in the center of the treatment area, and vacuum suction was initiated. The vacuum itself fixed the applicator over the treatment area, and pillows supported the applicator during the entire treatment.

### 2.5. Anthropometric Measurements

All the participants underwent anthropometric measurements that were performed at the baseline and during follow-ups 30, 60, and 90 days after the treatment. During assessments of their weight and height, the subjects wore only their underwear, without shoes. A classical mechanical stadiometer (model 110 CH, Welmy, São Paulo, Brazil) was used. The circumference of the abdomen was measured using a flexible measuring tape. Each measurement point was recorded at the baseline to ensure that subsequent measurements would be obtained from the same location.

### 2.6. Questionnaire

The patients completed a self-questionnaire that assessed the tolerability of the treatment. Volunteers indicated the tolerability of the treatment: selecting 1 for intolerable, 2 for tolerable, 3 for comfortable, and 4 for very comfortable.

### 2.7. Skinfold Caliper

A skinfold caliper (RMC, Amparo, SP, Brazil) was used to measure the site of greatest thickness within the treatment area for patients who were available for measurements at the baseline and during follow-ups 30, 60, and 90 days after the treatment.

### 2.8. Ultrasound Analysis

All the subjects underwent diagnostic ultrasound that was performed at the baseline and during follow-ups 30, 60, and 90 days after the treatment. Ultrasound assessment was performed using a linear transducer with a frequency of 6 to 18 MHz (MyLab™25 Gold; Esaote, Italy). Images were analyzed through quantitative measurements of the subcutaneous tissue between the anatomic planes—the dermis and muscular fascia—and the thickness of the fat layer at the treatment area was measured in millimeters [26]. A single trained physiotherapist made the measurements. The probe was positioned on the previously demarcated points in the treatment area (Figure 2), with coupling gel and without tissue compression.

### 2.9. Standardized Photographs

Standardized digital photographs were taken with a digital camera (Canon EOS Rebel T3i, Canon USA Inc., Melville, NY, USA) at the baseline and during a follow-up 90 days after the treatment. All patients were photographed in standing positions in 3 views: the back view, right view, and left view; the image was taken at a distance of 1 m.

### 2.10. Safety Assessments

The safety assessments included laboratory testing and adverse-event monitoring. Blood collections were performed at the baseline and between 14 and 21 days after treatment for evaluations of the fasting glucose levels, lipid profiles, and liver function. Blood samples for all subjects were collected via venipuncture on a morning after overnight fasting of 12 to 14 hours. Immediately after the collection, the samples were processed and analyzed in a laboratory—São Francisco Laboratório de Análises Clínicas (Amparo, SP, Brazil). Serum lipid values were obtained for the following elements: cholesterol, triglycerides, very-low-density lipoprotein cholesterol, low-density lipoprotein cholesterol, and high-density lipoprotein cholesterol. Liver-related blood tests were obtained through the evaluation of the hepatic markers aspartate aminotransferase and alanine aminotransferase. Subjects with baseline laboratory values outside the reference range were excluded from this analysis. The occurrence of adverse events was monitored throughout the study.

### 2.11. Statistical Analysis

The statistical analysis was performed with Graph Pad Prism 6 (La Jolla, CA, USA). The normal-distribution assumption was assessed with a Shapiro-Wilk test. The differences between the beginning and posttreatment measurements were analyzed using an ANOVA and Tukey's multiple comparisons test or Friedman test and Dunn's multiple-comparisons test. For comparisons of glucose, the serum-liver test and serum-lipid values from the baseline and 3 weeks after the treatment were analyzed using the paired* t*-test or Wilcoxon signed-rank test. The level of significance for all tests was set at 0.05 (*P* < 0.05).

## 3. Results

**Figure 3:**
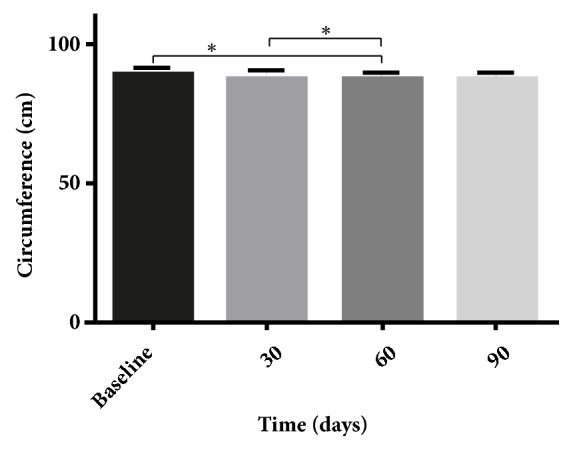
Means, with a standard error of the mean, of the waist circumference values before treatment (baseline) and after treatment (30, 60, and 90 days after treatment). ^*∗*^*P *< 0.05).

Twenty-one subjects were enrolled and completed treatment (18 females and 3 males). The subjects were aged from 21 to 50 years, with a mean and standard deviation of 34 ± 9 years. Their weights ranged from 57.5 to 90.5 kg, with a mean and standard deviation of 70.3 ± 9; their BMI ranged from 21.8 to 30.0, with a mean and standard deviation of 25.7 ± 2. Weight and BMI did not undergo significant changes after treatment. The waist circumference data are presented in Figure 3. The measurements from the baseline and 30-day follow-up had statistically significant differences, as did those from the 30-day and 60-day follow-ups. However, we have not found differences between the measurements from the 90-day follow-up and the baseline.

The subjects tolerated the treatment well: 35% (N = 8) of the volunteers reported that it was tolerable, and 60% (N = 12) reported that it was comfortable. Only 1 volunteer (5%) considered the treatment intolerable.

**Figure 4:**
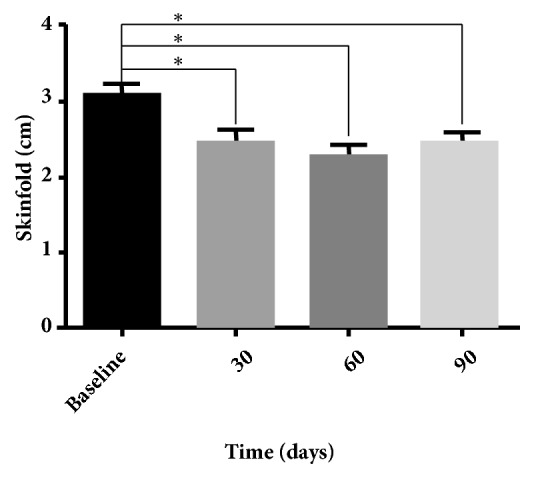
Means, with a standard error of the mean, of the abdominal length skinfold before treatment (baseline) and after treatment (30, 60, and 90 days after treatment). ^*∗*^*P* < 0.05.

The skinfold caliper data were analyzed to assess the treatment efficacy. The decrease in the skinfold-thickness values from the follow-ups and the baseline in the areas treated was statistically significant (*P* < 0.05) (Figure 4).

**Figure 5:**
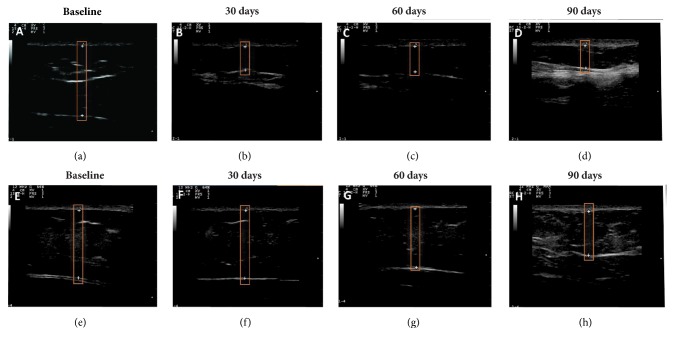
The thickness of the abdominal fat layer (a-d)—(a) at the baseline, (b) at the 30-day follow-up, (c) at the 60-day follow-up, and (d) at the 90-day follow-up—and the thickness of the flanks' fat layer (e-h): (e) at the baseline, (f) at the 30-day follow-up, (g) at the 60-day follow-up, and (h) at the 90-day follow-up. Note the hyperechoic areas—bright echoes and highly reflective structures (white = dermis fascia and fibrotic septa)—and hypoechoic areas: sparse echoes, reflection, or intermediate transmission (gray = adipose tissue and skeletal muscle). The boxes indicate the areas compared and the decrease of the thickness after the treatment.

Ultrasound images were analyzed to calculate the fat-layer reduction.  Figure 5 shows representative ultrasound images captured at the baseline and during follow-ups 30, 60, and 90 days after the treatment.

**Figure 6:**
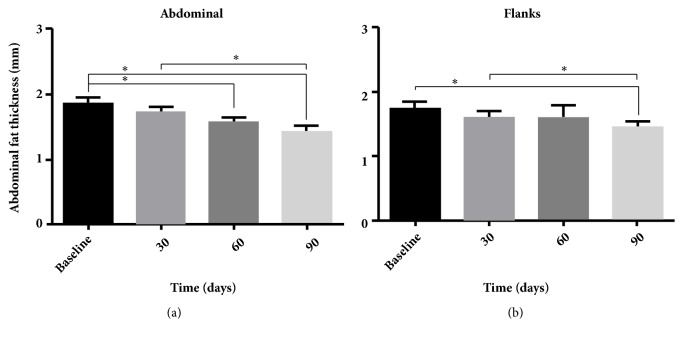
Means, with standard error of the mean, from an (a) abdominal fat-thickness assessment through diagnostic ultrasound before treatment (baseline) and after treatment (30, 60, and 90 days after treatment) and a (b) flank fat-thickness assessment through diagnostic ultrasound before treatment (baseline) and after treatment (30, 60, and 90 days after treatment). ^*∗*^*P* < 0.05.

The reductions in the fat layer were statistically significant (*P* < 0.05) in both treated regions: the abdomen and flanks. The mean percentage of fat-layer reduction was 21.6% for the abdomen, and reductions of up to 50.1% were detected from the baseline to 90 days after the treatment (Figure 6(a)). In the flanks, the mean reduction was 14.5%, and reductions of up to 43.2% were observed from the baseline to 90 days after the treatment (Figure 6(b)).

**Figure 7:**
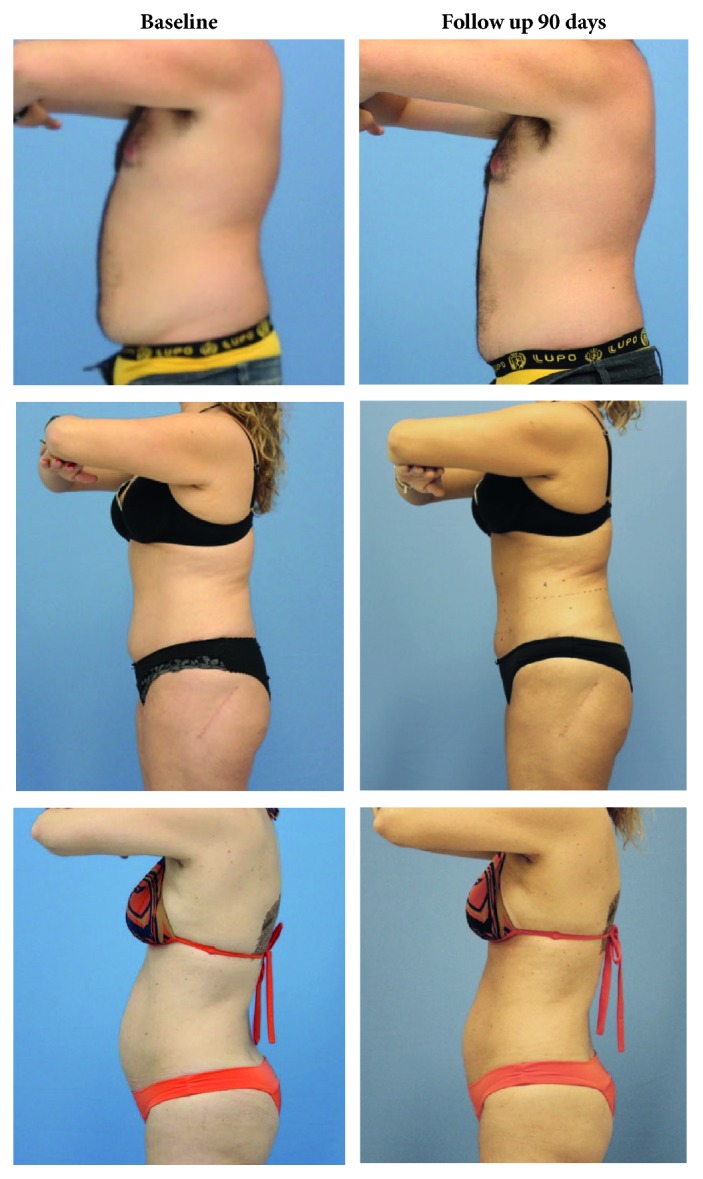
Comparison from the baseline and 90-day follow-up after the treatment with contrast cryolipolysis.

Figure 7 contains photographs that show that the sizes of the abdominal and flank areas visibly reduced between the baseline and 90-day follow-up.

The laboratory results are shown in Table 1. The mean values and standard deviations for the fasting glucose, liver-related tests, and serum lipids from the baseline were analyzed and compared with those from 3 weeks after the treatment. No significant changes were found.

## 4. Discussion

The purpose of the present study was to evaluate the safety and efficacy of contrast cryolipolysis: a method mixes the principles of conventional cryolipolysis and the contrast lipocryolysis process, which involves periods of heating.

The mechanism by which cryolipolysis induces damage to adipocytes is not well understood and remains an ongoing subject of research. The effectiveness and safety of this method has been widely established; however, it is important to highlight that the majority of published studies use a device that uses a cooling-intensity factor (CIF) and does not display the temperature in Celsius [27, 28]. The first study [29] with pigs that used a conventional cryolipolysis prototype were performed to investigate the effect of a controlled application of cold to the skin surface and the resulting selective damage to subcutaneous fat through the use of a cooled-fold metal plate. The skin surface areas were exposed to a cooling process with different temperatures (20°C, −1°C, −3°C, −5°C, and −7°C), and 20°C was considered the control temperature. The application time was 10 minutes. The authors observed that the apoptosis of fat cells started when the cooling panels were cooled to the temperature of −1°C. However, compared with the control, the most-intense results were obtained with the temperature of −7°C. In a subsequent study [30], also carried out with pigs, the researchers translated the temperature in degrees Celsius to milliwatts per centimeter squared and converted the result into CIF, a numerical value that defines heat extraction (cooling). The authors of this study observed a progressive inflammatory response to cold exposure. Immediately after treatment, no significant changes in subcutaneous fat were observed; however, 3 days after treatment, the presence of an inflammatory infiltrate was observed. The influx of inflammatory cells had increased when they were analyzed 7 and 14 days after treatment. After 30 days, the inflammatory process had begun to decline, and by 60 days, the thickness of the interlobular septa had apparently increased. The inflammatory process had weakened further by 90 days after treatment. The authors clearly showed that cryolipolysis induced subcutaneous panniculitis in response to cold exposure, with a decrease of the thickness of the fat layer, without affecting surrounding structures such as skin and water-rich tissue [24, 31]. These studies resulted in the development of the Coolsculpting™ device (Zeltiq Aesthetics, Inc., Pleasanton, CA) [30, 31]. Since then, the publications that have used this device [28] have used values in CIF to express the rate of heat extraction. It is difficult to draw comparisons between the results of studies in general and the specific results of studies using Coolsculpting. Publications [20, 32] only recently provided the values of the treatment temperature in degrees Celsius.

A study involving another line of research (lipocryolysis) [33]—which used the isolated adipocyte suspension of Wistar rats that were exposed to a temperature of 8°C for 0, 10, or 25 minutes—observed more crystallization when the exposure duration was increased. It was also observed that the crystals did not disappear when the samples were warmed at room temperature (22°C) for 2 hours. A subsequent study [12] applied several temperature patterns (heating and cooling) in isolated rat adipocytes and observed an increase in the crystallization process when using contrast temperatures: heating-cooling-heating. Both processes, cooling and contrast temperatures, showed high potential to induce the cellular death of adipocytes through lipid crystallization [11, 33]. This research contributed to the development of the Lipocryo® technology (Clinipro, S.L. Barcelona, Spain), and it is important to highlight that the technique of contrast lipocryolysis uses the extraction of temperatures up to 3°C [34].

A case report of a patient who went through a cryolipolysis treatment at −5°C with the Galeno device (South Korea) demonstrated, through diagnostic ultrasound, a reduction in the thickness of the fat layer. Histological analyses of the material collected during a later abdominoplasty showed significant adipocyte destruction [27].

In a study performed by Sasaki et al. [35], which used the Coolsculpting device, they inserted a temperature probe into the subcutaneous tissue in the treated area and revealed that the temperature in the tissues reached as low as 9°C.

Because studies use different nonsurgical devices that control cooling to decrease subcutaneous fat without damaging the surrounding tissues and each of them uses temperatures that range from the CIF index to 3°C, it is difficult to compare studies.

One fact is clear: cold-induced lipid crystallization (crystal-structure formation) of the adipocytes occurs at temperatures around 8°C to 10°C, and it is a condition dependent on time and temperature; this seems to be the key to the results [11–13, 33, 35].

In our study, all the participants were treated with a Polarys® device (Ibramed, Amparo, SP, Brazil) using the following protocol: initial heating of 40°C for 10 minutes, temperature extraction for 60 minutes (a cooling temperature of −8°C set in the device), and heating to 40°C for 10 minutes at the end of the cooling cycle. Extracting the temperature from the skin creates a thermal gradient. The superficial layers (skin surface) become cooler and deepen into the subcutaneous tissue (adipose layer) as the treatment proceeds [35]. The procedures were performed using vacuum-pressure applicators (medium or large size) for heating and extracting heat from both sides of a fold and reducing blood flow via tissue compression and cold-induced vasoconstriction. The total time of the treatment was 80 minutes for each area treated. Each subject received treatment in 3 to 5 areas: the abdomen or flanks, according to individual's needs. The treatment was performed in 1 or 2 visits.

During the treatment period, no significant changes in body weight or BMI occurred. However, compared to the baseline measurements, the waist circumferences were significantly reduced at the 30-day and 60-day follow-ups; the circumferences also decreased between the 30-day and 60-day follow-ups. No differences in waist circumference were observed at the 90-day follow-up (Figure 3). Despite the methodological accuracy, we believe that some factors may have affected the measurements at the 90-day follow-up [36]. This result contrasts measurements obtained through skinfold caliper (Figure 4) and diagnostic ultrasound (Figures 5-6).

The cold-induced metabolic stress during the treatment and the production of reactive oxygen species during the postischemia reperfusion that occurs immediately after the removal of the applicator have been the target of studies [37, 38]. From the beginning, in both preclinical and clinical studies, a cycle of massage (1-5 minutes) was applied immediately after procedure to facilitate the homogeneity of crystallization in the treatment area [29, 30, 37, 38]. It is believed that the process of reperfusion, after cooling of the tissue, can generate an increase in reactive oxygen species, the cytosolic calcium, and the activation of dependent and independent calcium proteolytic enzymes, including caspases that activate apoptotic pathways [35, 39]. In our study, the application time was 10 minutes of heating up to 40°C to increase reperfusion. This could explain our good results compared to those of other clinical studies that used ultrasound imaging to measure the abdominal fat layer. The averages for the fat-thickness reduction in these studies were 18.2% [24], 19.5% [28], and 19.6% [35] measured 6 months after the treatments. In our study, the mean percentage of fat-layer reduction was 21.6%, measured 3 months after treatment (Figure 6(a)). Reductions of up to 50.1% were detected at the baseline and after 3 months. The results were also effective in the flanks area; the mean percentage of reduction was 14.5%, and reductions of up to 43.2% were observed in comparisons of the measurements from the baseline and 3-month follow-up (Figure 6(b)). The results can be observed in the comparative photographs in Figure 7.

In terms of safety, we did not note any significant impact on fasting glucose, lipid levels, or liver function tests (aspartate aminotransferase and alanine aminotransferase) after contrast cryolipolysis treatments, as seen in Table 1. These results are similar to those obtained by Klein et al. [37], who used conventional cryolipolysis; the destruction of adipocytes does not significantly affect serum-lipid levels or liver-function tests.

In this study, 84 areas were treated with contrast cryolipolysis: 54 areas were treated with a medium applicator, and 30 areas were treated with a large applicator. The subjects reported mild to moderate discomfort at the treated site, especially during the contrast phase; however, the treatment was considered tolerable by 95% of the sample. The typical side effects of cryolipolysis procedures reported in clinical studies include erythema, edema, bruising, and transient neuralgia [40]. Subjects in this study experienced temporary adverse effects—such as redness, slight bruising, and numbness—that resolved spontaneously. The subjects did not report pain from neuralgia or burns.

## 5. Conclusion

Contrast cryolipolysis was safe and effective in the treatment of localized fat in the flanks and abdominal region. Even though the study was performed with a number of individuals greater than the number suggested by the sample calculation, we believe that studies with a larger sample should be performed.

## Figures and Tables

**Table 1:** Mean (SD) for fasting glycemia, serum liver test, and serum lipid values.

**Analysis (units)**	**Reference**	**Time**		***P* value**
		**Baseline**	**3 weeks later**	
Glucose (mg/dL)^a^	70-100	85.71 ± 9.04	84.64 ± 10.99	0.862

AST (U/L)^a^	10 - 37 ♀ / 11 - 39 ♂	18.96 ± 5.83	21.27 ± 5.90	0.169

ALT (U/L)^a^	11 - 45 ♀ / 10 - 37 ♂	17.67 ± 11.96	21.27 ± 8.71	0.136

Cholesterol (mg/dL)^a^	< 200	176.4 ± 36.35	166.1 ± 25.54	0.491

Triglycerides (mg/dL)^b^	< 200	103 ± 63.16	89.18 ± 57.42	0.765

HDL Cholesterol (mg/dL)^a^	> 65	50.65 ± 12.01	47.09 ± 7.22	0.348

LDL Cholesterol (mg/dL)^a^	< 130	105.2 ± 30.49	101.2 ± 26.45	0.443

VLDL Cholesterol (mg/dL)^b^	< 40	20.59 ± 12.63	17.84 ± 11.48	0.765

ALT, alanine aminotransferase; AST, aspartate aminotransferase; HDL, high-density lipoprotein; LDL, low-density lipoprotein; VLDL, very-low-density lipoprotein.

^a^Paired *t*-test.

^b^Wilcoxon signed-rank test.

A *P*-value < 0.05 is considered statistically significant.

## Data Availability

The data used to support the findings of this study are available from the corresponding author upon request.
